# Malaria-related hospitalization during childhood in Papua, Indonesia: A retrospective cohort study

**DOI:** 10.1371/journal.pone.0228018

**Published:** 2020-01-29

**Authors:** Nicholas M. Douglas, Enny Kenangalem, Afdhal Hasanuddin, Nicholas M. Anstey, Paulus Sugiarto, Ric N. Price, Jeanne Rini Poespoprodjo

**Affiliations:** 1 Global and Tropical Health Division, Menzies School of Health Research, Charles Darwin University, Darwin, Australia; 2 Division of Infectious Diseases, Royal Darwin Hospital, Darwin, Australia; 3 Timika Malaria Research Program, Papuan Health and Community Development Foundation, Timika, Papua, Indonesia; 4 Mimika District Health Authority, Timika, Papua, Indonesia; 5 Department of Paediatrics, Rumah Sakit Mitra Masyarakat, Timika, Papua, Indonesia; 6 Centre for Tropical Medicine and Global Health, Nuffield Department of Clinical Medicine, University of Oxford, Oxford, United Kingdom; 7 Mahidol-Oxford Tropical Medicine Research Unit, Faculty of Tropical Medicine, Mahidol University, Bangkok, Thailand; 8 Department of Child Health, Faculty of Medicine, Public Health and Nursing, Universitas Gadjah Mada, Yogyakarta, Indonesia; 9 Department of Paediatrics, Rumah Sakit Umum Daerah, Kabupaten Mimika, Timika, Papua, Indonesia; Instituto Rene Rachou, BRAZIL

## Abstract

**Background:**

In endemic regions, the age distribution of malaria varies according to the infecting *Plasmodium* species. We aimed to delineate the pattern of malaria-related hospitalization from birth in Timika, Papua–an area co-endemic for *P*. *falciparum* and *P*. *vivax*

**Methods:**

Between April 2004 and December 2013, infants born at Mitra Masyarakat Hospital, or presenting within the first 7 days of life, were enrolled retrospectively into a cohort study and followed passively using routinely-collected hospital surveillance data. Outcomes were stratified by the presence or absence of *Plasmodium* parasitemia and included re-presentation to hospital, requirement for hospital admission and death.

**Results:**

Overall, 11,408 infants were enrolled into the cohort. Median follow-up was 4.3 (maximum 9.7) years. In total, 7,847 (68.9%) infants made 90,766 re-presentations to hospital, 18,105 (19.9%) of which were associated with *Plasmodium* parasitemia. The incidence of re-presentations with malaria during the first year of life was 213 per 1,000 person-years (py) for *P*. *vivax* and 79 per 1,000py for *P*. *falciparum* (Incidence Rate Ratio (IRR) = 2.69, 95% Confidence Interval (95%CI): 2.48–2.92). After the age of 5 years, the incidence of *P*. *vivax* had fallen to 77/1,000py and the incidence of *P*. *falciparum* had risen to 95/1,000py (IRR = 0.80, 95%CI: 0.73–0.88). Overall, 79.7% (14,431/18,105) of malaria re-presentations were recurrences rather than initial infections. Malaria accounted for 31.7% (2,126/3,120) of all hospital admissions. The infant mortality rate in this study was 52 deaths per 1,000 live births. Beyond the early neonatal period, 13.4% of deaths were associated with *Plasmodium* parasitemia.

**Conclusions:**

In Papua, Indonesia, malaria is a major cause of hospital presentation and admission in early life. The initial predominance of *P*. *vivax* over *P*. *falciparum* inverts after five years of age. Malaria is directly associated with nearly one in seven deaths after the early neonatal period.

## Introduction

Malaria is caused by mosquito-borne, protozoan parasites of the genus *Plasmodium*. The most common species globally are *P*. *falciparum* and *P*. *vivax* [1]. In malaria endemic regions, the age distribution of symptomatic and/or severe *Plasmodium* infections is determined primarily by the force of infection and the speed with which immunity develops. For both epidemiological and biological reasons (most notably the ability of *Plasmodium vivax* to relapse multiple times after a single inoculation), the age distribution frequently differs for *P*. *falciparum* and *P*. *vivax* malaria [2–7].

Acute malaria in children causes a wide spectrum of clinical manifestations ranging from an uncomplicated febrile illness to severe disease and death. Recurrent episodes of malaria during early life have deleterious effects on health as well as causing substantial social and economic hardship for children and their families [8–11]. Malaria in endemic regions can also exact a large toll on hospital resources with attendant opportunity costs. Determining the pattern of symptomatic *Plasmodium* infections in non-study settings requires an ability to differentiate malaria from other causes of febrile illness coupled with an ability to link multiple clinical episodes to a single individual.

The aim of this study was to use routinely collected hospital data to delineate the pattern of malaria-related morbidity and mortality from birth in a cohort of children in Timika, Papua. We also aimed to define the proportionate contribution of malaria care to the total hospital workload for these children.

## Materials and methods

This was a retrospective cohort study of children who were either born at Mitra Masyarakat Hospital (RSMM) in Timika, Papua between April 2004 and December 2013 or whose first presentation to hospital during this accrual period occurred within the first 7 days of life. Follow-up information for these children was obtained passively from routinely-collected hospital data via unique hospital identification number linkage, as described previously [12,13].

### Site

Timika is situated in the lowlands of southcentral Papua, Indonesia. The population in 2004 was 120,457 growing to 196,401 in 2013 [14]. The main ethnic groups in the region are Highland and Lowland Papuans and Indonesians from elsewhere in the country. Until late 2009, RSMM was the only referral hospital in the district. After that date, a second public hospital opened (RSUD). RSMM has 110 inpatient beds, a high care unit and a 24-hour emergency department. Both the outpatients and the emergency departments are open every day and together attend to approximately 100,000 patient presentations per year. Healthcare at RSMM is provided at a small cost to non-Papuan Indonesians (3–5 USD for outpatient malaria treatment), but is free of charge to Papuans from one of the 7 largest local tribes, who make up 93% of attendees. As such the hospital is the preferred source of medical care for inpatient, outpatient and antenatal encounters [15]. A community household survey of treatment seeking behavior in 2005, estimated that more than 80% of children under 5 who had died in the preceding year had done so at the RSMM hospital [16]. Malaria transmission in Timika is perennial with minimal seasonal variation. The main mosquito vectors are *Anopheles punctulatus*, *An*. *koliensis* and *An*. *farauti* complex. The point prevalence of microscopic parasitemia in 2005 was 7.5% for *P*. *falciparum* and 6.4% for *P*. *vivax* with an estimated incidence of malaria overall of 876 episodes per 1,000 people per year [16]. In 2013 the point prevalence was 5.2% for *P*. *falciparum* and 5.7% for *P*. *vivax* [17]. Malaria diagnostics and treatment can be sought at RSMM (and, since January 2010, at RSUD), one of the 12 government-funded community public health clinics, one of 10 clinics administered by the malaria control arm of the PT Freeport Indonesia mining corporation, or at a range of unregulated private sector facilities such as shops and pharmacies. Vector control activities in the region were fairly consistent between 2002 and 2013 and involved twice-yearly indoor residual spraying and distribution of insecticide-treated bednets–covering approximately 10–20% of households [13].

### Hospital data collection and procedures

Data for every patient presentation to RSMM are collected routinely and entered into an electronic Q-pro^®^ database by hospital administrators. Information recorded includes patient identifiers (with a unique hospital number assigned at the point of first presentation to hospital), demographic details such as age, sex and ethnicity, clinical information including hospital department, diagnoses assigned by the attending physician (using the International Classification of Diseases 10 coding system) and vital status at the end of the clinical episode.

Hospital protocol dictates that a blood sample is taken for Giemsa staining and thick film examination for any patient presenting to outpatients with fever or history of fever and all patients who are severely ill, regardless of presumed diagnosis. Microscopy in early neonates was not routine during the study period and only requested if there was clinical concern. If parasitemia is too high to count on a thick film, the hospital microscopists will also read a thin film.

Prior to April 2006, treatment of uncomplicated malaria at RSMM generally consisted of oral quinine for *P*. *falciparum* infections and oral quinine plus low-dose primaquine (3.5mg/kg total dose) for *P*. *vivax* and *P*. *ovale* infections. The treatment of severe malaria during this era was IV quinine. In April 2006, treatment policy was changed to dihydroartemisinin-piperaquine for uncomplicated malaria due to any *Plasmodium* species with the addition of high dose primaquine (7mg/kg total dose) for *P*. *vivax* and *P*. *ovale* infections. At the same time, treatment of severe malaria was also changed to intravenous artesunate [18].

### Outcomes and data analysis

Patients aged less than 7 days old at their first appearance in the hospital dataset were selected and followed passively until the 31^st^ of December 2013 by means of unique hospital identification number linkage. Outcomes of interest were: subsequent re-presentation to hospital (defined as any new attendance at the hospital after study entry), admission for inpatient care, total bed occupancy (assuming 0.5 days of occupancy for outpatient and emergency department visits) and death during the hospital encounter with delineation of the absolute and proportionate contributions of *P*. *falciparum*, *P*. *vivax* and mixed *Plasmodium* species infections to these outcomes. Patients with no further documented re-presentation to hospital were assumed to have remained in the hospital catchment area and therefore to have been at risk of re-presentation until the 31^st^ of December 2013. For the purposes of graphical presentation, actuarial life tables were used to determine the number of patients at risk of the outcomes of interest in each 6-month period from birth. Kaplan-Meier survival analysis was used to estimate the cumulative risk of the outcomes of interest and incidence rates were given per 1,000 person-years for the two age brackets <1 year and ≥5 years. The effect of previous hospital presentation on the risk of a further representation was explored using Kaplan-Meier survival analysis and univariable and multivariable Cox regression models for each *Plasmodium* species. Covariables in the multivariable Cox models included: age, sex, ethnicity (Highland Papuan, Lowland Papuan, non-Papuan) and the year of birth. The proportional hazards assumption was assessed for each variable by comparing visually observed versus predicted survival curves. When the proportional hazards assumption was violated, Cox models were either stratified or omitted altogether. Allowance was made in all Cox models for within-patient correlation by using the Huber-White sandwich estimator to calculate robust standard errors. All analyses and graphing were done in STATA^®^ version 15.1 (College Station, Tx).

### Ethics approval and consent to participate

Ethical approval for this study was obtained from the Health Research Ethics Committees of the University of Gadjah Mada, Indonesia (KE/FK/544/EC) and Menzies School of Health Research, Darwin, Australia (HREC 10.1397). Data were anonymized prior to access for this study. The ethics committees waived the requirement for informed consent and therefore individual patient consent for participation was not sought.

## Results

Between April 2004 and December 2013, 11,408 neonates were enrolled into the cohort, of whom 52.4% (n = 5,974) were male. In total 48.9% (5,559) were Highland Papuan, 20.1% (2,290) were lowland Papuan and 31.0% (3,526) were non-Papuan. Of these patients, 11,307 (99.1%) either did not have a malaria diagnostic test or were negative for *Plasmodium* parasitemia at the point of entry to the study and 101 (0.90%) had congenital malaria. The median duration of follow-up from the time of entry to the study until the 31^st^ of December 2013 was 4.3 years and the maximum follow-up was 9.7 years.

### Total hospital usage

**Fig 1:**
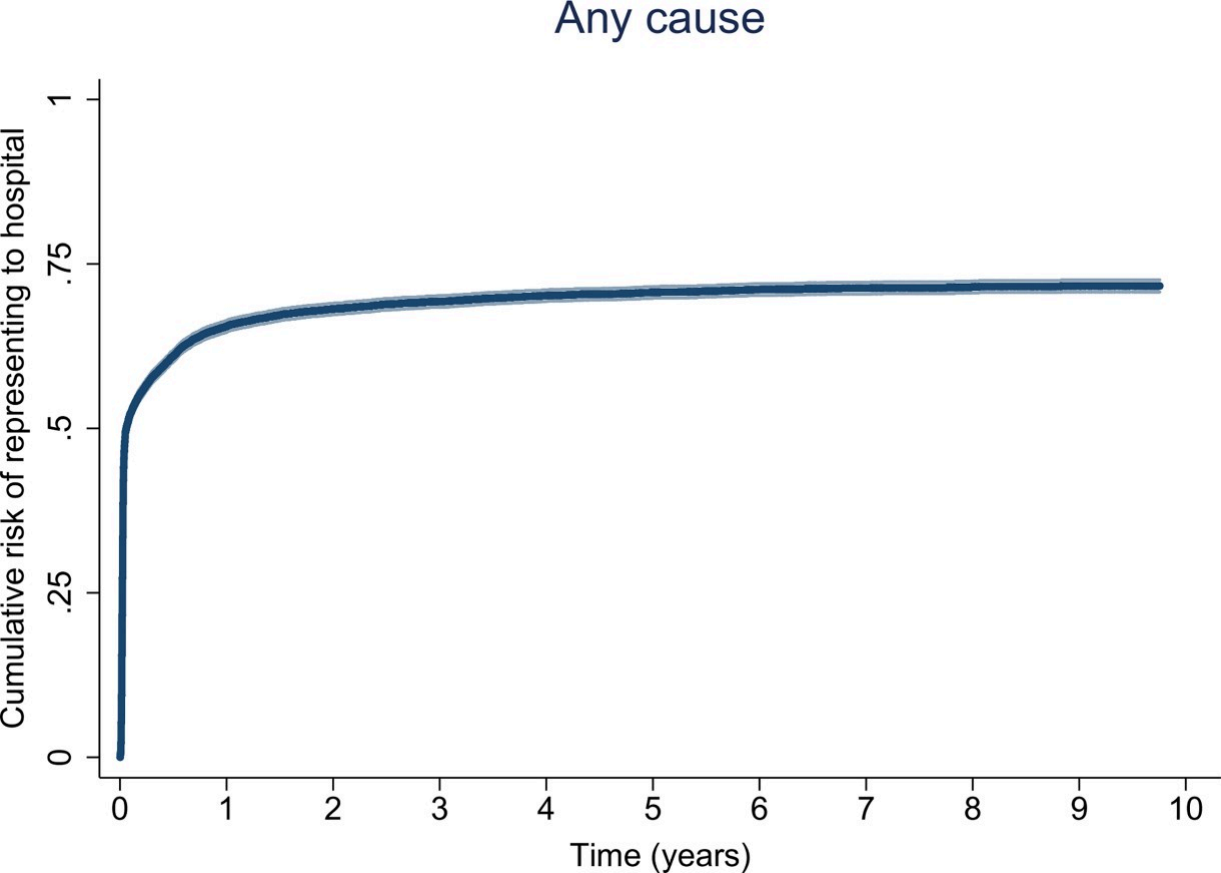
Cumulative risk of first re-presentation to hospital due to any cause by time from birth (with 95% confidence interval).

Overall 7,847 (68.8%) patients re-presented 90,766 times to hospital during follow-up. By 5 years of age the cumulative risk of at least one re-presentation was 70.7% (95% Confidence Interval (95%CI) 69.8–71.6%) with a median number of 9 presentations (maximum = 134) (**Fig 1**). In total 93.6% (7,343/7,847) of first re-presentations occurred within the first 12 months of life (cumulative risk = 65.5%, 95%CI 64.7–66.4%).

**Fig 2:**
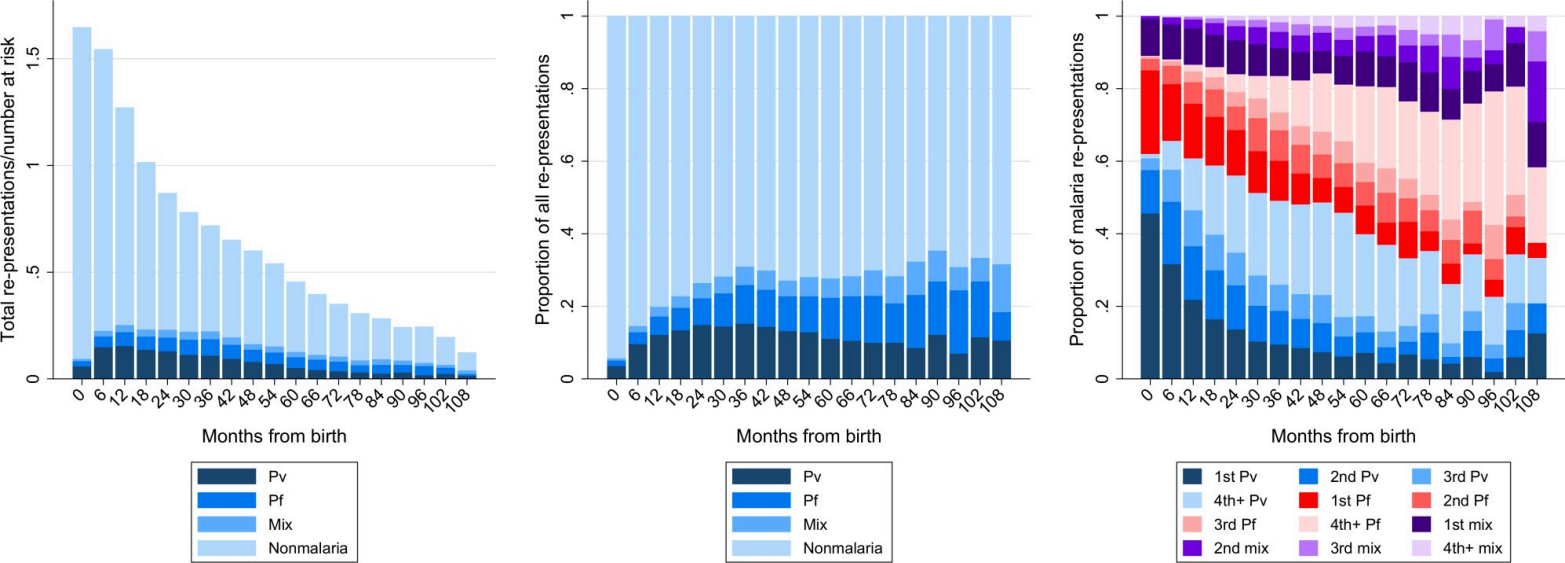
**Total number of re-presentations divided by number of patients at risk (left) and proportion of all re-presentations by age stratified by *Plasmodium* species (middle).** Proportion of malaria re-presentations due to the different *Plasmodium* species stratified by the number of previous re-presentations due to the same species (right). Abbreviations: Pv; *Plasmodium vivax*, Pf; *Plasmodium falciparum*, Mix; mixed *Plasmodium* species infection.

Overall 19.9% (18,105/90,766) of representations were attributable to malaria with a cumulative risk of at least one malaria re-presentation by 5 years of 38.1% (95%CI 37.1–39.1%); **Fig 2**. Of the malaria-related re-presentations, 5,462 (30.2%) were due to *P*. *falciparum*, 9,556 (52.8%) to *P*. *vivax*, 6 (0.0%) to *P*. *ovale*, 200 (1.1%) to *P*. *malariae* and 2,881 (15.9%) to mixed species infection.

In the first year of life, the incidence of malaria was 335 events per 1,000 person-years (py). The incidence of *P*. *vivax* infections (213/1,000py) was significantly higher than that of *P*. *falciparum* infections (79/1,000py; Incidence Rate Ratio (IRR) = 2.69, 95%CI: 2.48–2.92). After the age of 5 years the overall incidence of malaria declined to 225 events per 1,000py (IRR = 0.67, 95%CI: 0.64–0.71)) with a reduction in the incidence of *P*. *vivax* to 77 per 1,000py (IRR = 0.35, 95%CI: 0.33–0.38), but an increase in *P*. *falciparum* to 95/1,000py (IRR = 1.19, 95%CI: 1.09–1.31). Within the first year of life, 49.9% (1,699/3,402) of malaria-related presentations were first episodes whereas after the age of 5 years, only 6.0% (137/2,292) of presentations were first episodes. The corresponding *Plasmodium* species specific proportions due to first episodes were 75.3% and 15.9% for *P*. *falciparum* and 55.9% and 16.4% for *P*. *vivax*. Overall, 79.7% (14,431/18,105) of the presentations with malaria were recurrences rather than initial infections.

**Fig 3:**
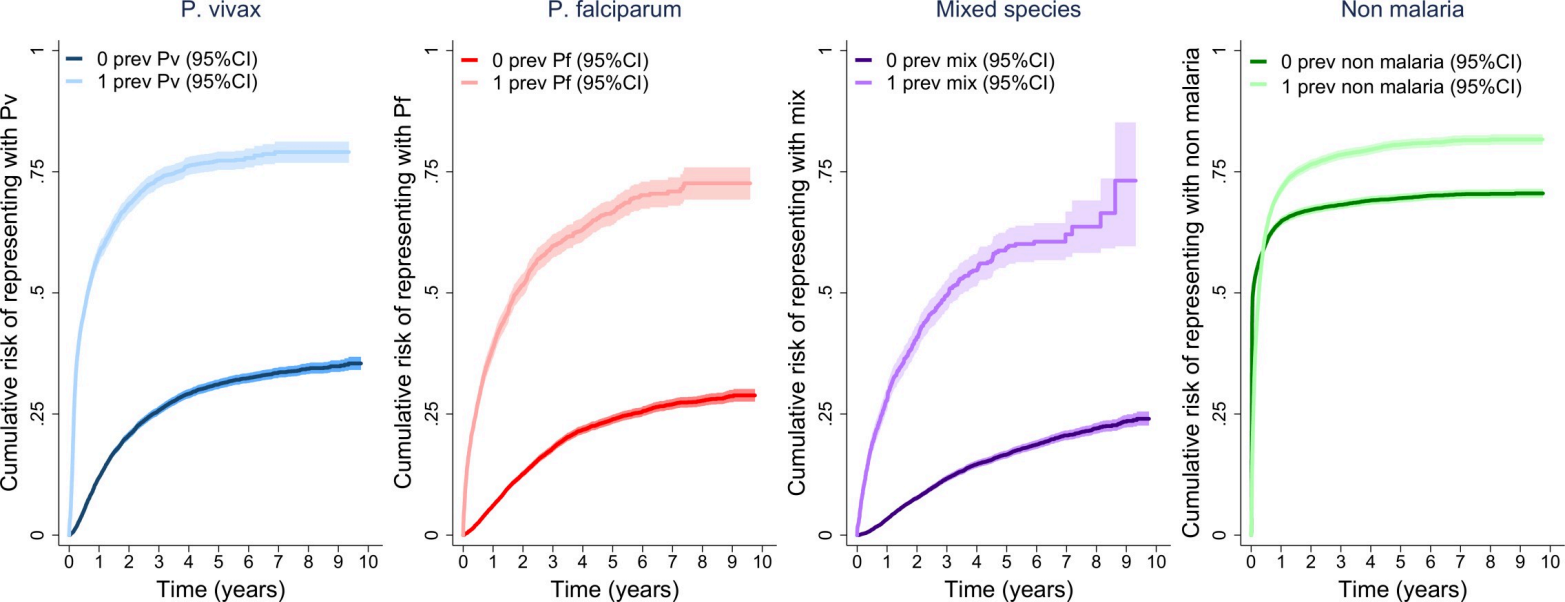
Kaplan-Meier curves showing the cumulative risk of re-presentation with a recurrent episode due to the same *Plasmodium* species. Abbreviations: prev; previous, Pv; *Plasmodium vivax*, Pf; *Plasmodium falciparum*, mix; mixed *Plasmodium* species infection, 95%CI; 95% confidence interval.

Previous presentation to hospital with malaria was a major risk factor for further re-presentation with malaria (Hazard Ratio (HR) = 4.8, 95%CI 4.5–5.0, p<0.001) and this was still apparent after controlling for confounding factors (Adjusted Hazard Ratio (AHR) = 3.0, 95%CI 2.8–3.2, p<0.001; Table 1). This finding was similar for *P*. *vivax* monoinfections (AHR = 3.2, 95%CI 2.9–3.4, p<0.001) and *P*. *falciparum* monoinfections (AHR = 2.8, 95%CI 2.6–3.1, p<0.001; **Fig 3** and Table 1). Conversely, prior presentation with a nonmalarial illness was not associated with a substantially increased risk of a further nonmalarial re-presentation: the 5-year cumulative risk of re-presentation being 80.6% (95%CI: 79.6–81.6%) versus 69.6% (95%CI: 68.7–70.4%) respectively (violation of proportional hazards precluded Cox regression). Highland Papuans were at significantly greater risk of re-presentation with malaria than both Lowland Papuans and non-Papuans (Table 1).

**Table 1:** Cox regression models showing the effect of a previous episode of malaria on the risk of re-presenting with a further episode of malaria, stratified by *Plasmodium* species.

	*Plasmodium vivax*						*Plasmodium falciparum*						Mixed *Plasmodium* species					
	Univariable			Multivariable^a^			Univariable			Multivariable^b^			Univariable			Multivariable^a^		
	HR	95%CI	p	AHR	95%CI	p	HR	95%CI	p	AHR	95%CI	p	HR	95%CI	p	AHR	95%CI	p
**Previous episode caused by same species**																		
No	Ref			Ref			Ref			Ref			Ref			Ref		
Yes	5.32	(5.01–5.66)	<0.001	3.17	(2.92–3.43)	<0.001	5.09	(4.72–5.48)	<0.001	2.84	(2.56–3.14)	<0.001	5.98	(5.43–6.59)	<0.001	2.60	(2.27–2.98)	<0.001
**Ethnicity**																		
Non-Papuan	Ref			Ref			Ref			Ref			Ref			Ref		
Highland Papuan	14.7	(12.3–17.6)	<0.001	11.0	(9.22–13.1)	<0.001	14.4	(11.8–17.6)	<0.001	10.5	(8.63–12.8)	<0.001	43.4	(29.0–64.7)	<0.001	33.9	(22.7–50.5)	<0.001
Lowland Papuan	4.55	(3.73–5.56)	<0.001	4.01	(3.31–4.85)	<0.001	5.43	(4.34–6.79)	<0.001	4.70	(3.80–5.82)	<0.001	14.5	(9.53–22.1)	<0.001	13.2	(8.70–19.9)	<0.001
**Sex**																		
Male	Ref			Ref			Ref			Ref			Ref			Ref		
Female	0.97	(0.91–1.04)	0.409	0.98	(0.92–1.04)	0.429	0.97	(0.89–1.05)	0.388	0.96	(0.89–1.03)	0.213	0.99	(0.90–1.09)	0.867	1.00	(0.92–1.08)	0.945
**Age (per year increase)**	1.42	(1.39–1.46)	<0.001	1.03	(1.00–1.07)	0.046	1.48	(1.44–1.52)	<0.001				1.48	(1.45–1.52)	<0.001	1.07	(1.03–1.11)	0.001

^a^ Model stratified by year of birth

^b^ Model stratified by year of birth and age group (<1 year, 1–5 years, >5 years)

Abbreviations: ref, reference category, HR; hazard ratio, AHR; adjusted hazard ratio, 95%CI; 95% confidence interval

### Hospital admission

**Fig 4:**
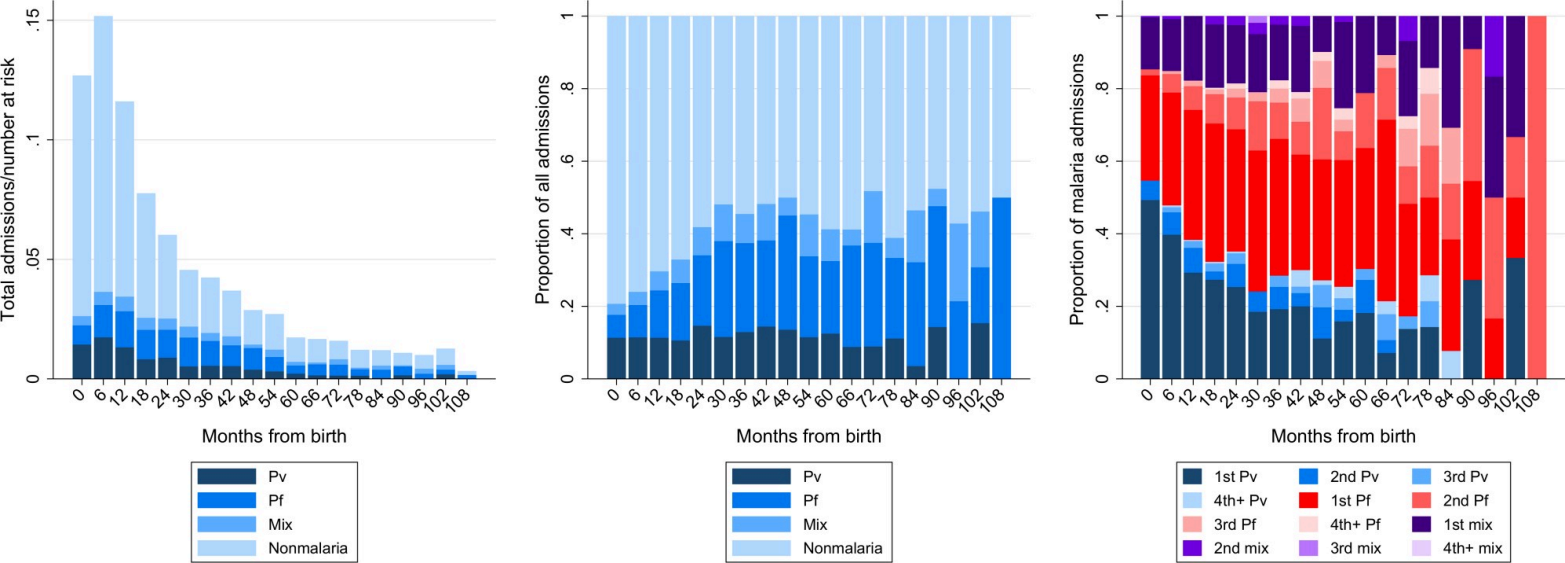
**Total number of admissions divided by number of patients at risk (left) and proportion of all admissions by age from birth stratified by *Plasmodium* species (middle).** Proportion of malaria admissions due to the different *Plasmodium* species stratified by the number of previous admissions due to the same species (right). Abbreviations: Pv; *Plasmodium vivax*, Pf; *Plasmodium falciparum*, Mix; mixed *Plasmodium* species infection.

Overall, 3,120 children had 6,710 admissions to hospital during follow-up. The 5-year cumulative risk of at least one admission was 31.1% (95%CI: 30.2–32.1%), with 48.6% (1,517/3,120) of these patients being admitted more than once (median = 2, maximum = 20). Malaria due to any *Plasmodium* species was present in 2,126 (31.7%) of the children admitted (**Fig 4**). Within the first year of life, malaria was present in 22.6% (675/2,990) of inpatients whereas after the age of 5 years, the proportion with malaria had risen to 43.9% (133/303) of admissions. The incidence of malaria admissions was 67/1,000py in children under one year of age and 14/1,000py after the age of 5 years (IRR = 4.80, 95%CI: 4.00–5.79). The incidence of *P*. *vivax*-related admissions decreased from 34/1,000py in infants under one year to 3/1,000py (IRR = 11.0, 95%CI: 7.62–16.5) whereas there was a smaller reduction in the incidence of *P*. *falciparum* admissions from 23/1,000py to 8/1,000py (IRR = 2.87, 95%CI: 2.21–3.75).

Overall, 11.9% (353/2,967) of children presenting for the first time with a *P*. *vivax* monoinfection required admission to hospital whereas 20.9% (458/2,187) of initial *P*. *falciparum* presentations resulted in admission. Inpatient care of the children in this cohort resulted in 26,604 days of bed occupancy, of which 6,586 days (24.8%) were in patients with malaria: 4,149 days (15.6%) in patients admitted for the first time with malaria and 2,437 days (9.2%) in patients admitted for the second or subsequent time with malaria. The proportion of patients being admitted for the second or subsequent time with the same species was higher with *P*. *vivax* in the first 18 months, but higher with *P*. *falciparum* thereafter (**Fig 4**).

### Hospital deaths

**Fig 5:**
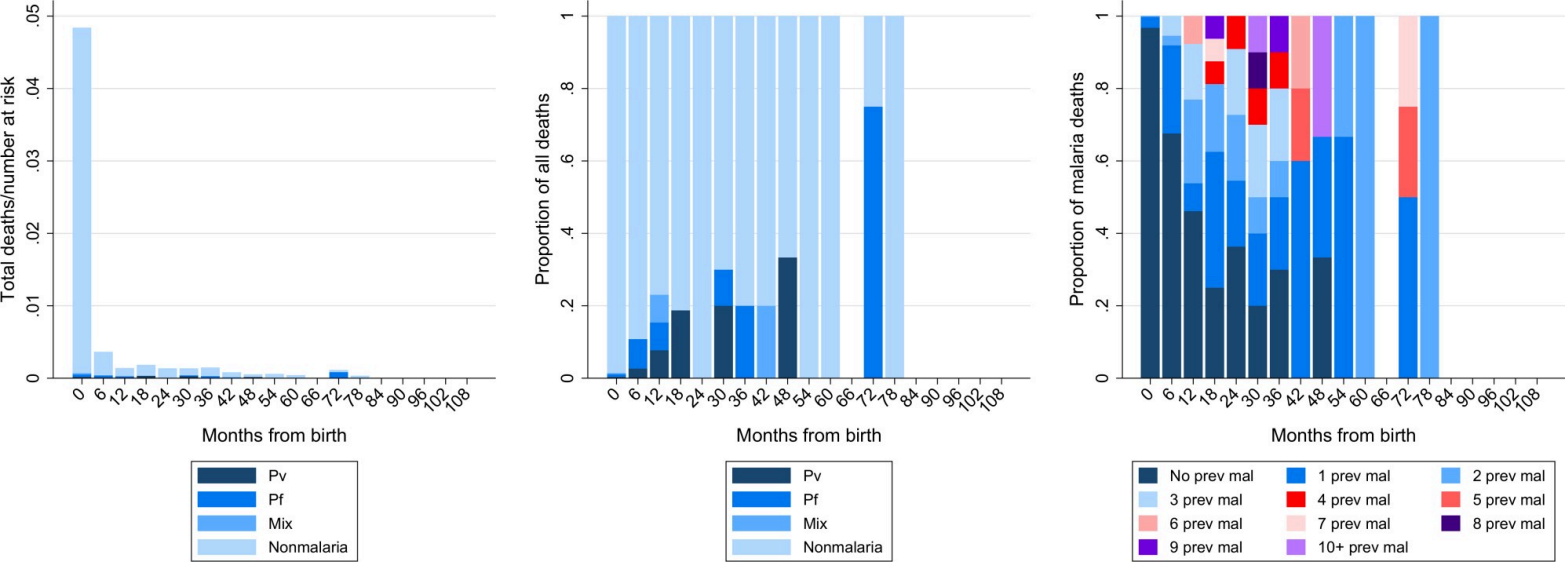
**Total number of deaths divided by number of patients at risk (left) and proportion of all deaths by age from birth and *Plasmodium* species (middle).** Proportion of malaria deaths due to the different *Plasmodium* species stratified by the number of previous malaria episodes (right). Abbreviations: Pv; *Plasmodium vivax*, Pf; *Plasmodium falciparum*, Mix; mixed *Plasmodium* species infection, Mal; malaria.

In total 667 children in the cohort died at the hospital of whom 480 (72.0%) were less than 7 days old (early neonatal deaths), 481 (72.1%) were less than 28 days old (neonatal mortality rate = 42 deaths per 1,000 live births) and 589 (88.3%) were less than one year old (infant mortality rate = 52 deaths per 1,000 live births) (**Fig 5**). The cumulative risk of death by one year was 5.3% (95%CI 4.9–5.7%) and by 5 years this had risen to 6.1% (95%CI 5.7–6.6%). Overall 4.3% (29/667) of the patients had a diagnosis of malaria at the time of death: 14 (48.3%) due to *P*. *falciparum*, 10 (34.5%) due to *P*. *vivax* and 4 (13.8%) due to mixed species infection. Beyond the early neonatal period, 13.4% (25/187) of deaths were associated with *Plasmodium* parasitemia. The cumulative risk of a malaria related death was 0.1% (95%CI 0.07–0.21) by one year and 0.3% (95%CI 0.20–0.44) by 5 years.

## Discussion

We present a large, retrospective, hospital-based birth cohort study in southern Papua, Indonesia. Over 70% of the children in the study re-presented to the hospital during a median of 4.3 years of follow-up and almost 40% re-presented with malaria. Malaria accounted for about 20% of all hospital re-presentations, 32% of hospital admissions and 25% of total inpatient bed occupancy. The incidence of *P*. vivax-related hospital presentations decreased significantly between infancy and 5 years of age whereas the incidence of *P*. *falciparum* increased. Prior malaria, whether due to *P*. *vivax* or *P*. *falciparum*, was a strong risk factor for a further presentation with malaria. Indeed 80% of all malaria-related presentations made by the children in this study were recurrences rather than initial infections. Despite the major contribution of malaria to the total hospital workload, direct mortality associated with malaria was dwarfed by the high early neonatal mortality in this cohort (approximately 1 in 24 live births). While patent parasitemia was only present in 4.3% of all children who died in hospital, this rose to 13.4% among those who died after day seven.

Given that a single episode of malaria, regardless of species, identifies a child as being at significant risk for future re-presentation with malaria, the first episode of malaria represents a particularly important opportunity to intervene to prevent morbidity and mortality from multiple recurrences and substantially reduce hospital workload. Some of the increased risk of recurrence will be attributable to *P*. *vivax* relapses, preventable, in those who are eligible, by using radically curative courses of primaquine [19–21]. Unsupervised primaquine has previously been shown to be relatively ineffective for preventing re-presentation to hospital with vivax malaria in this region, likely due to poor medication adherence [12]. Supervision of therapy and/or primaquine regimens that encourage better adherence compared with the existing 14-day course would be highly desirable. Our finding that *P*. *falciparum* infection also increases the risk of malaria recurrence suggests that at least part of the increased risk may be due to epidemiological risk factors such as socio-economic status, proximity to mosquito breeding areas and local vector control activity. Geolocating patients presenting for the first time with malaria may be an effective strategy to target transmission hotspots through vector control, distribution of insecticide treated bednets and/or mass drug administration. Current vector control strategies in the region are targeted at village, rather than individual, level. A third explanation for the increased risk of re-presentation in those with prior malaria is simply that an initial malaria presentation identified a subset of patients who were more likely to seek treatment for any condition at RSMM than the remaining children–potentially due to proximity or specific health care preferences. Given that previous nonmalaria-related presentations had relatively little bearing on the risk of a future re-presentation, this is unlikely to have been the sole explanation.

Ideally, testing for glucose-6-phosphate dehydrogenase (G6PD) deficiency should be undertaken prior to prescription of primaquine for radical cure of *P*. *vivax* infection to minimise the risk of drug-induced hemolysis. One of the barriers to primaquine use is the cost of such testing [22]. Our results suggest that the number of G6PD tests required at a facility such as RSMM would be much lower than the total number of *P*. *vivax* presentations might suggest. Nearly 85% of presentations with vivax malaria by the age of 5 years are recurrences as opposed to first episodes and G6PD status need only be assessed once.

Although presentation to hospital with malaria was dominated by recurrent infections rather than primary infections, requirement for admission to hospital (presumably reflecting severe disease or an inability to take oral medication) occurred mainly in patients presenting for the first time with malaria. The risk was almost twice as high in children presenting with falciparum malaria, a likely reflection of poor immunity and susceptibility to pathogenic processes. We have previously hypothesized that morbidity (particularly severe anemia) caused by vivax malaria tends to result from the cumulative effects of repeated infections rather than a single infection [23,24]. Approximately a fifth of admissions in patients with vivax malaria were in children who had previously presented to hospital with vivax malaria, a slightly lower proportion than for falciparum malaria. This suggests that: 1) initial vivax infections can cause sufficiently severe manifestations to warrant admission to hospital, 2) admission in those with vivax malaria is mostly due to concomitant morbidities or 3) a significant proportion of preceding, less-complicated, episodes of vivax malaria are managed in the primary care system outside the hospital. As shown previously, the incidence of *P*. *vivax* infection was heavily skewed towards children under the age of one year whereas *P*. *falciparum* infections predominated after the age of 5 years [4]. This likely reflects more rapid acquisition of immunity to *P*. *vivax* due to greater parasite exposure resulting from multiple relapses [25].

Perhaps the most striking finding in this study was the very high infant mortality rate of 52 deaths per 1,000 births, largely attributable to early neonatal deaths. In 2017 the mean infant mortality rate globally was 29 deaths per 1,000 live births, rising to 51 deaths per 1,000 live births in Africa [26]. Anecdotally, approximately 90% of the babies in this study were delivered in hospital electively to women with pregnancies not deemed to be high risk. The remaining 10% of mothers had high risk pregnancies and had been referred to hospital to deliver. The risk of neonatal mortality after delivery in the latter group of women would almost certainly have been higher than in the community and therefore the estimates of childhood mortality will have been somewhat inflated. Regardless, infant mortality in this region remains very high.

Our study has some notable strengths. Due to the lengthy accrual period, relatively large local population and the hospital’s busy obstetric unit, our study included a large number of children and therefore our risk estimates are relatively precise. The hospital’s strict policy regarding blood film assessment ensured that the vast majority of patients with patent parasitemia presenting to the hospital will have been captured. RSMM was the only public tertiary referral centre in the district until late 2009 and therefore a very high proportion of all pediatric inpatient admissions and deaths in hospitalized patients will have been ascertained. Beyond 2009, some admissions and deaths may have occurred at the other public hospital even though birth occurred at RSMM.

Our study has some important limitations. Patients presenting with malaria to community health care facilities (or, after December 2009, to the other public hospital in the region) and those who migrated out of the catchment area prior to the study conclusion will not have been captured. Any ascertainment bias will have underestimated the risk of re-presentation and the true burden of symptomatic malaria in our cohort. This bias was likely to have been particularly important for non-Papuan patients who were required to pay a small amount for health care at RSMM. Our survival analyses of the risk of further re-presentation to hospital only included a relatively limited number of covariables. Data on household income and bednet usage were not available and may have influenced the risk of malaria recurrence. Attribution of events to malaria was based on the presence or absence of patent parasitemia. Some individuals may have had symptomatic subpatent parasitemia while others may have had patent parasitemia that was incidental to other comorbidities. Moreover, previous episodes of malaria may have caused morbidity that contributed to nonmalarial re-presentations. Congenital malaria was frequent in the early years of the study [27], prior to ACT policy change, but microscopy at birth was not routine during this time. While the proportion of early neonatal deaths with parasitemia is likely to be small, a direct contribution from malaria cannot be excluded in this period. Malaria is also well recognised as a significant indirect cause of mortality [28,29], and the indirect contribution to early neonatal mortality (for example by causing preterm birth and low birth weight [30]) and post-neonatal mortality could not be measured. Finally, follow-up of the children in this study ended on the 31^st^ of December 2013. Population growth, variations in treatment seeking behaviour and changes in the epidemiology of malaria in the region will have altered the pattern of malaria presentations at RSMM in the last 5 years.

## Conclusions

Malaria is a very common cause of representation to hospital during childhood in southcentral Papua, resulting in significant morbidity, mortality and hospital workload. Malaria tends to be recurrent after the initial presentation making the first occurrence a particularly important opportunity to implement preventive measures through optimal antimalarial treatment and geospatially targeted vector control activities. Despite malaria being directly associated with nearly one in seven deaths after the neonatal period, early neonatal causes of mortality predominate in this setting and warrant prospective investigation and intervention.

## Supporting information

S1 Data Study data in excel format. (XLSX)
